# X-linked inhibitor of apoptosis protein accelerates migration by inducing epithelial–mesenchymal transition through TGF-β signaling pathway in esophageal cancer cells

**DOI:** 10.1186/s13578-019-0338-3

**Published:** 2019-09-18

**Authors:** Yuxiang Jin, Xinye Lu, Mingdong Wang, Xuewei Zhao, Lei Xue

**Affiliations:** Department of Thoracic Surgery, Changzheng Hospital, Second Military Medical University, 415 Fengyang Road, Shanghai, 200003 People’s Republic of China

**Keywords:** Esophageal cancer cells, X-linked inhibitor of apoptosis protein, Migration, Epithelial–mesenchymal transition, Transforming growth factor-β

## Abstract

**Background:**

The prognosis of esophageal cancer is still dismal because of its high probability of metastasis that is likely related to the cellular process of epithelial–mesenchymal transition (EMT). Recent studies have shown a novel role of X-linked inhibitor of apoptosis protein (XIAP) in regulating the migration process of cancer cells and, therefore, linking to progression and poor prognosis of cancer.

**Methods:**

The expression of XIAP in esophageal squamous cell cancer (ESCC) tissues was determined by immunohistochemistry assay. Cell migration was analyzed by wound healing assay and Transwell assay. The expression of EMT markers (E-cadherin, N-cadherin and Vimentin) was revealed by immunofluorescence assay. Quantitative real‑time PCR analysis and Western blot analysis were used to detect the expression of XIAP and EMT markers as well as transforming growth factor-β (TGF-β) at mRNA and protein level, respectively.

**Results:**

We found that the expression of XIAP closely correlated to the probability of lymphatic metastasis in patients and that ESCC patients with the high XIAP expression were associated with worse overall survival (OS). Univariate and multivariate analysis also revealed XIAP as an independent prognostic factor for overall survival in ESCC patients. In both EC9706 and TE13 cell lines, knockdown of XIAP decreased the migration of cancer cells by inhibiting EMT process through regulating the TGF-β signaling pathway, pinpointing a regulatory role of XIAP in migratory process upon TGF-β activation.

**Conclusions:**

Taken together, our results suggest XIAP as a important prognostic and regulative factor in ESCC patients. XIAP may promote migration of esophageal cancer cells through the activation of TGF-β mediated EMT.

## Introduction

Esophageal cancer which ranks the sixth cause of death among all cancers is one of the most aggressive tumors worldwide with high morbidity and poor prognosis [1]. Since esophageal cancer is asymptomatic in early stage, most patients are diagnosed at late stages which limit curative treatment [2]. Among which 70% have obvious local lymph node infiltration and metastasis and lose the chance of surgery, only 30% of patients have the opportunity to receive radical surgery, but even after surgery, the prognosis of this malignancy is still dismal because of high probability of migration and metastasis [3]. Therefore, defining a prognostic factor for early diagnosis of esophageal cancer is not only important to understand the molecular mechanisms responsible for malignant development and progression, but also provide new therapeutic targets to improve the survival rate.

Inhibitor of apoptosis proteins (IAPs) are potentially involved in multiple cellular signaling pathways including cell death, cell cycle, cell migration, immunity, inflammation [4, 5]. X-linked inhibitor of apoptosis protein (XIAP) is a key member of the IAP family that was first identified by Rajcan-Separovic in 1996 [6]. XIAP is a 497 amino acid protein composed of three BIR domains, one UBA domain and one RING domain, whose genes are located in the Xq25 region of the X chromosome [7]. In addition, XIAP is known as a direct inhibitor of proteolytic caspase activity by specifically inhibiting caspases 3, 7, and 9 via its three BIR domains [8, 9]. High level expression of XIAP has been detected in various kind of cancers and has been shown to relate to chemoresistance, progression of disease and poor prognosis [10–12].

Epithelial–mesenchymal transition (EMT) is a cellular transformation from epithelial to mesenchymal phenotype, which enables cancer cells more efficiently invade and migrate [13]. In the progress of cancer development, EMT is considered as an important process in the induction of tumorigenesis, invasion, metastasis, stemness and resistance to therapy as well as poor clinical outcome [14, 15]. E-cadherin is a calcium-dependent transmembrane glycoprotein and typically expresses in most epithelial cells, while N-cadherin and Vimentin have been consistently associated with mesenchymal transition [16, 17]. Therefore, down-regulation of E-cadherin and up-regulation of N-cadherin and Vimentin are considered as indication of the occurrence of EMT process that was found to positively correlate with cancer metastasis [18].

In the current study, we examined the expression of XIAP in ESCC patients and its relationship with prognosis. We further examined the potential mechanisms by which XIAP facilitates EMT process in cancer cell lines. These findings determined a novel role of XIAP in ESCC and represents a potential target for ESCC therapy.

## Methods

### Patients and tissue samples

Tissue samples were obtained from 185 ESCC patients who underwent esophagectomy at Changzheng Hospital Affiliated to Second Military Medical University. The patients received preoperative radiotherapy or chemotherapy were excluded. Overall survival (OS) was defined as the interval between surgery and death or the last observation taken. Informed consent was obtained from each patient and this study was approved by the Ethics Committee of Changzheng Hospital.

### Cell lines

ESCC cell lines EC9706, TE13 were obtained from Shanghai Sunbio Company. All cells were cultivated in Dulbecco’s modified Eagle’s medium (DMEM, Gibco) supplemented with 10% fetal bovine serum (Gibco) at 37 °C in 5% CO_2_.

### Wound healing assay

Cell motility was assessed by wound-healing assay. Cells were seeded on 6-well dish and grown to 80% confluence. Monolayer was then disrupted with a 200 μL pipette tip, and images were captured at 0 and 48 h in a phase-contrast microscope. All experiments were carried out in triplicate.

### Transwell assay

Tumor cell migration assays were performed using a Transwell chamber with 8 µm pores in 24-well dishes. The cells (1 × 10^5^) were seeded in the upper chamber without serum, and the medium supplemented with 5% FBS was added in the lower chamber as a chemoattractant. The plates were incubated at 37 °C for 24 h, then the cells were fixed with 4% formaldehyde, stained with crystal violet dye and counted under a light microscope.

### Western blotting

TE13 and EC9706 cells were lysed in radio immunoprecipitation assay (RIPA) buffer containing protease inhibitors. Equal amounts of proteins were separated by SDS-PAGE and transferred to polyvinylidene fluoride (PVDF) membranes. Then the membranes were sealed with 5% non-fat milk for 2 h at room temperature. After overnight incubation with primary antibody, the membrane was washed and then incubated with secondary antibodies for 2 h. At last, the targeted blots were visualized by the enhanced chemiluminescence substrate and detected by ECL system (Tanon, China). The primary antibodies used in our study including XIAP (1:1000, NO.14334, CST, USA), E-cadherin (1:2000, NO.ab53226 Abcam, USA), N-cadherin (1:1000, NO.ab18203, Abcam, USA), Vimentin (1:1000, NO.ab92547, Abcam, USA), TGF-β (1:1000, NO.3711s, CST, USA), GAPDH (1:5000, NO.HRP-60004, Proteintech, USA). Secondary antibodies were goat anti-mouse antibody (1:5000, NO.SA00001-1, Proteintech, USA), goat anti-rabbit antibody (1:5000, NO.SA00001-2, Proteintech, USA).

### Immunofluorescence assay

Cells were plated on a 48-well dish. After 24 h, the cells were washed with phosphate-buffered saline (PBS) and then fixed with 4% formaldehyde and permeabilized with 0.1% Triton X-100. Thereafter, the cells were blocked with 1% bovine serum albumin (BSA) and incubated with primary antibodies overnight at 4 °C. Next, the cells were washed by PBS and incubated with secondary antibody at room temperature for 1 h, followed by counterstaining with DAPI for 10 min. All matched samples were observed under immunofluorescent microscopy. The primary antibodies were E-cadherin (1:500, NO.ab53226, Abcam, USA), N-cadherin (1:200, NO.ab18203, Abcam, USA), Vimentin (1:200, NO.ab92547, Abcam, USA). The secondary antibody was goat anti-rabbit antibody (1:1000, NO.A11011, Thermo Fisher Scientific, USA).

### Immunohistochemistry assay

The tissues were routinely fixed, dehydrated, paraffin embedded and sliced into sections (5 μm in thickness). Thereafter, the slides were processed with xylene, rehydrated and dehydrated, heat-induced antigen retrieval and blocked with 5% BSA. Thereafter, the slides were incubated with XIAP primary antibody (1:100, NO.14334, CST, USA) overnight at 4 °C, followed by incubation with secondary antibody for 30 min. Then the percentage of XIAP expression were scored according to intensity of staining and the percentage of positive cells (0: no staining; 1: ≤ 10%; 2: 10% to 50%; 3: ≥ 50%). The expression level of XIAP was divided into two groups according to score: low (0, 1), and high (2, 3).

### Quantitative real‑time PCR (qRT‑PCR)

Total RNAs were extracted from cells with TRizol Reagent (Invitrogen). Then converted into cDNA using Reverse Transcription Kit (Invitrogen). Expression of mRNA was determined by qRT‑PCR using SYBR Green Master Mix (Applied Biosystems, Foster City, CA, USA). Glyceraldehyde-3-phosphate dehydrogenase (GAPDH) was used as an internal control. The primer sequences used in qRT‑PCR were shown in Table 1.

**Table 1:** Primers used for qRT-PCR

Gene	Forward primer sequence (5′–3′)	Reverse primer sequence (5′–3′)
XIAP	AATAGTGCCACGCAGTCTACA	CAGATGGCCTGTCTAAGGCAA
Ecadherin	ATTTTTCCCTCGACACCCGAT	TCCCAGGCGTAGACCAAGA
Ncadherin	AGCCAACCTTAACTGAGGAGT	GGCAAGTTGATTGGAGGGATG
TGF-β	CAATTCCTGGCGATACCTCAG	GCACAACTCCGGTGACATCAA
Vimentin	AGTCCACTGAGTACCGGAGAC	CATTTCACGCATCTGGCGTTC
GAPDH	ACAACTTTGGTATCGTGGAAGG	GCCATCACGCCACAGTTTC

### Generation of stable XIAP knockdown cell lines

Recombinant lentiviral vectors was obtained from Obio Technology Co.Ltd. (Shanghai, China). The resultant plasmid was sequenced to confirm the authenticity of the insert. Cells were infected for 24 h with fresh lentiviral vectors expressing either vector control (Sh-Ctrl) or Sh-XIAP construct in medium. Next, cells were maintained in medium containing puromycin (2 μg/mL) for additional 72 h. The level of XIAP mRNA and protein expression in Sh-Ctrl and Sh-XIAP constructs was analyzed by Western blotting (Additional file 1: Figure S1).

#### ESCC cell lung metastatic assay

All animal experiments were approved by the Medical Experimental Animal Care Commission in Second Military Medical University. Mice at age of 6 weeks were randomly divided into two groups, EC9706 (sh-Ctrl) and EC9706 (sh-XIAP) were injected into nude mice via an I.V. lateral tail vein injection (2 × 10^6^ cells in 200 µL Physiological saline/mouse) respectively. The mice were sacrificed and lung nodules were counted at indicated times.

### Statistical analysis

Patient’s data were collected and analyzed by using the SPSS19.0 statistical software. The data were expressed as mean ± SD, and the comparative analysis between the two groups were performed by using unpaired *t* test. The categorical variables were expressed as frequencies and analyzed by using χ^2^ test. Kaplan–Meier analysis was used to evaluate the patient survival between two groups. Univariate analysis and multivariate analysis were used to test independent prognostic factors for overall survival. The difference was considered as statistically significant when p < 0.05.

## Results

### Correlation between XIAP expression and clinical characteristic

**Fig. 1:**
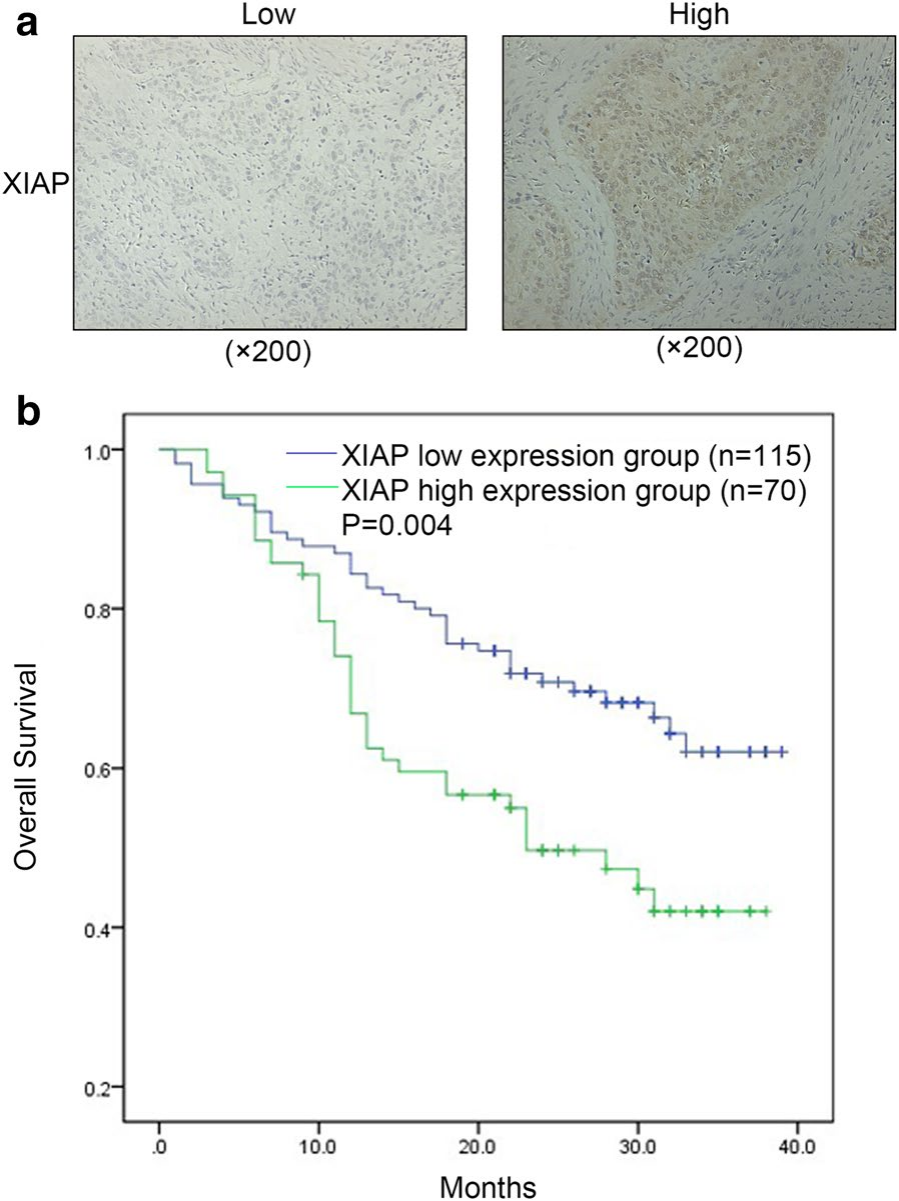
High expression of XIAP predicted poor prognosis in ESCC patients. **a** Low XIAP expression was seen in 115/185 (upper left ×200), and high in 70/185 of ESCC tissues (upper right ×200) by using IHC staining. **b** Estimated overall survival according to the expression of XIAP in 185 cases of ESCC, Kaplan–Meier method showed that ESCC patients in the high XIAP expression group had poorer overall survival than those in the low XIAP expression group (p = 0.004)

All 185 HCC patients were divided into two sub-groups according to the intensity of XIAP expression: low expression group (n = 115), and high expression group (n = 70) (Fig. 1a). To further explore the role of XIAP in the development and progression of ESCC, the relationship between XIAP expression and clinical characteristics was analyzed and tabulated in Table 2. The intensity of XIAP expression significantly correlated to the occurence of lymphatic metastasis (p = 0.018), while there were no statistical differences between XIAP expression and other clinical characteristics. Kaplan–Meier analysis demonstrated that patients with high expression of XIAP exhibited worse overall survival (OS) compared with the low expression group (p = 0.004, Fig. 1b). In univariate analysis, lymphatic metastasis and XIAP expression showed a significant association with poor overall survival (p = 0.001 and p = 0.005 respectively, Table 3). Multivariate analysis also revealed that lymphatic metastasis and XIAP expression were independent prognostic factors for overall survival in ESCC patients (p = 0.007 and p = 0.028 respectively, Table 4). The relationship between XIAP expression and EMT markers expression was shown in Table 5. The results showed a negative correlation between XIAP and E-cadherin expression (r = − 0.278, p < 0.001) and a positive correlation between XIAP and N-cadherin (r = 0.309, p < 0.001) and Vimentin (r = 0.209, p = 0.006) expression in ESCC tissues (Table 5).

**Table 2:** Correlation of XIAP expression with clinicopathological features of ESCC patients

Variable	XIAP expression (case)		p-value
	Low	High	
Gender			
Male	75	45	1.000
Female	40	25	
Age			
≤ 60	37	24	0.872
> 60	78	46	
Differentiation			
Well and medium	105	61	0.455
Poor	10	9	
T-stage			
T1–2	43	25	0.876
T3	72	45	
N-stage			
N0	90	43	0.018*
N1–2	25	27	
p-TNM stage			
I–II	93	49	0.107
III	22	21	

(* p < 0.05)

**Table 3:** Overall survival of ESCC patients: univariate analysis

Variable	HR	95% CI	p-value
Gender	0.841	0.531–1.331	0.459
Age	0.976	0.603–1.579	0.921
Differentiation	1.566	0.804–3.050	0.187
T-Stage	1.620	0.984–2.667	0.058
N-Stage	2.133	1.345–3.384	0.001*
XIAP	1.910	1.213–3.008	0.005*

(* p < 0.05)

**Table 4:** Overall survival of ESCC patients: multivariate analysis

Variable	HR	95% CI	p-value
N-Stage	1.906	1.188–3.058	0.007*
XIAP	1.684	1.058–2.681	0.028*

(* p < 0.05)

**Table 5:** Correlation between XIAP expression levels and EMT markers expression in ESCC tissues

	XIAP		p-value	r
	Low	High		
E-cadherin				
Low	64	58	0.000*	− 0.278
High	51	12		
N-cadherin				
Low	79	26	0.000*	0.309
High	36	44		
Vimentin				
Low	77	32	0.006*	0.209
High	38	38		

(* p < 0.05)

### XIAP knockdown inhibited migration of ESCC cells

**Fig. 2:**
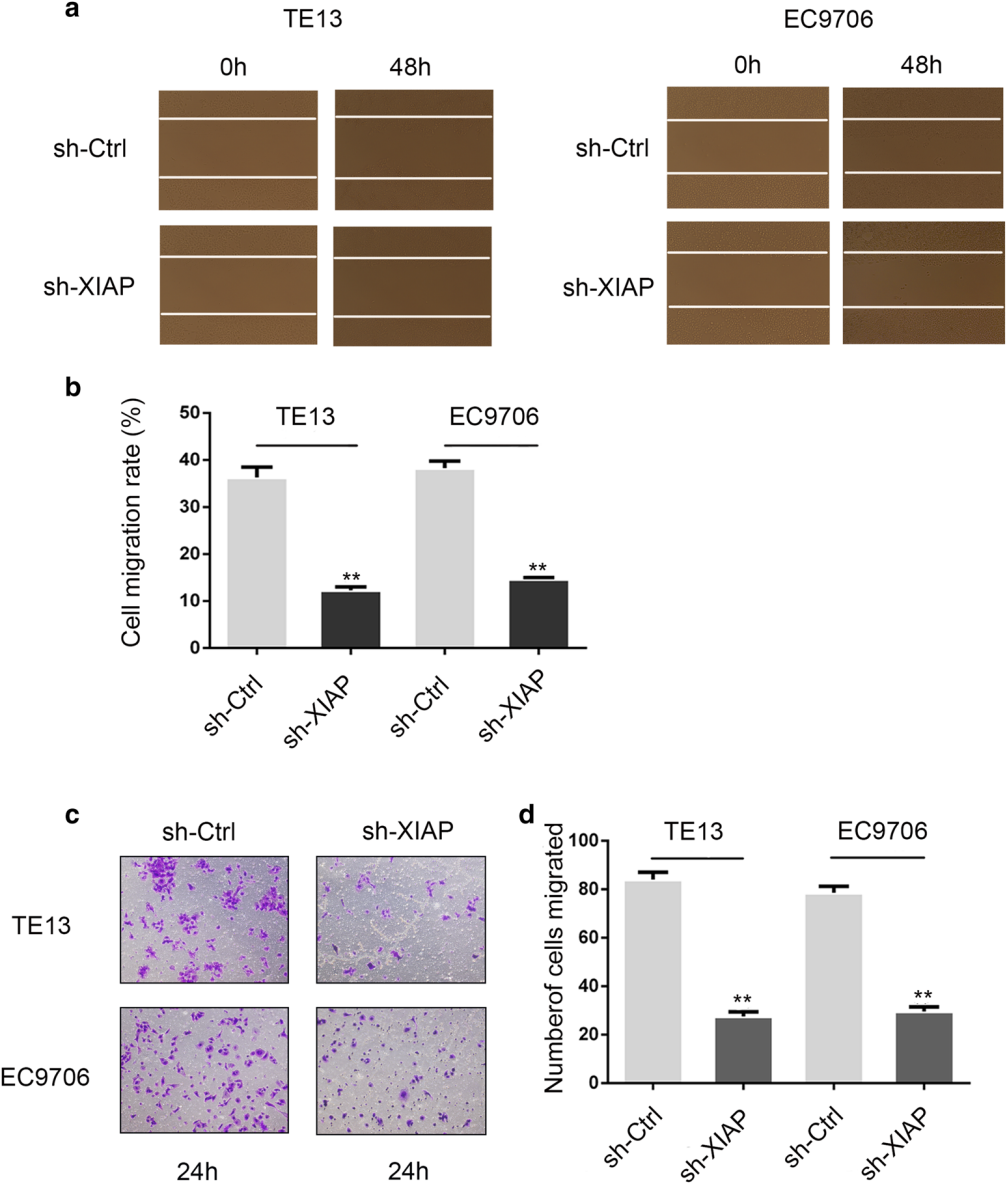
XIAP promoted the migration of ESCC cells. **a**, **b** The wound healing assay was employed to determine the migration of EC9706 and TE13 cells, cells were monitored for 48 h to determine the rate of migration into the scratched area (*p < 0.05, **p < 0.01). **c**, **d** The migration capacity of EC9706 and TE13 cells for 24 h was determined by Transwell assay. Number of cells that migrated through the Matrigel was counted in 10 fields (*p < 0.05, **p < 0.01)

Migration of cancer cells is a key step of cancer metastasis, thus we evaluated the cell migration in EC9706 and TE13 cells. As shown in Fig. 2a, b, cells in the sh-Ctrl groups exhibited marked cell migration in the wound area after 48 h wounding, while the sh-XIAP cells showed relative delays in wound closure (p < 0.01). By Transwell migration assay, the results showed an decrease in cell migration was observed in sh-XIAP groups compared with sh-Ctrl groups (Fig. 2c, d) (p < 0.01). This finding suggested that XIAP knockdown could effectively inhibit the migration ability of ESCC cells.

### XIAP knockdown suppressed EMT of ESCC cells

**Fig. 3:**
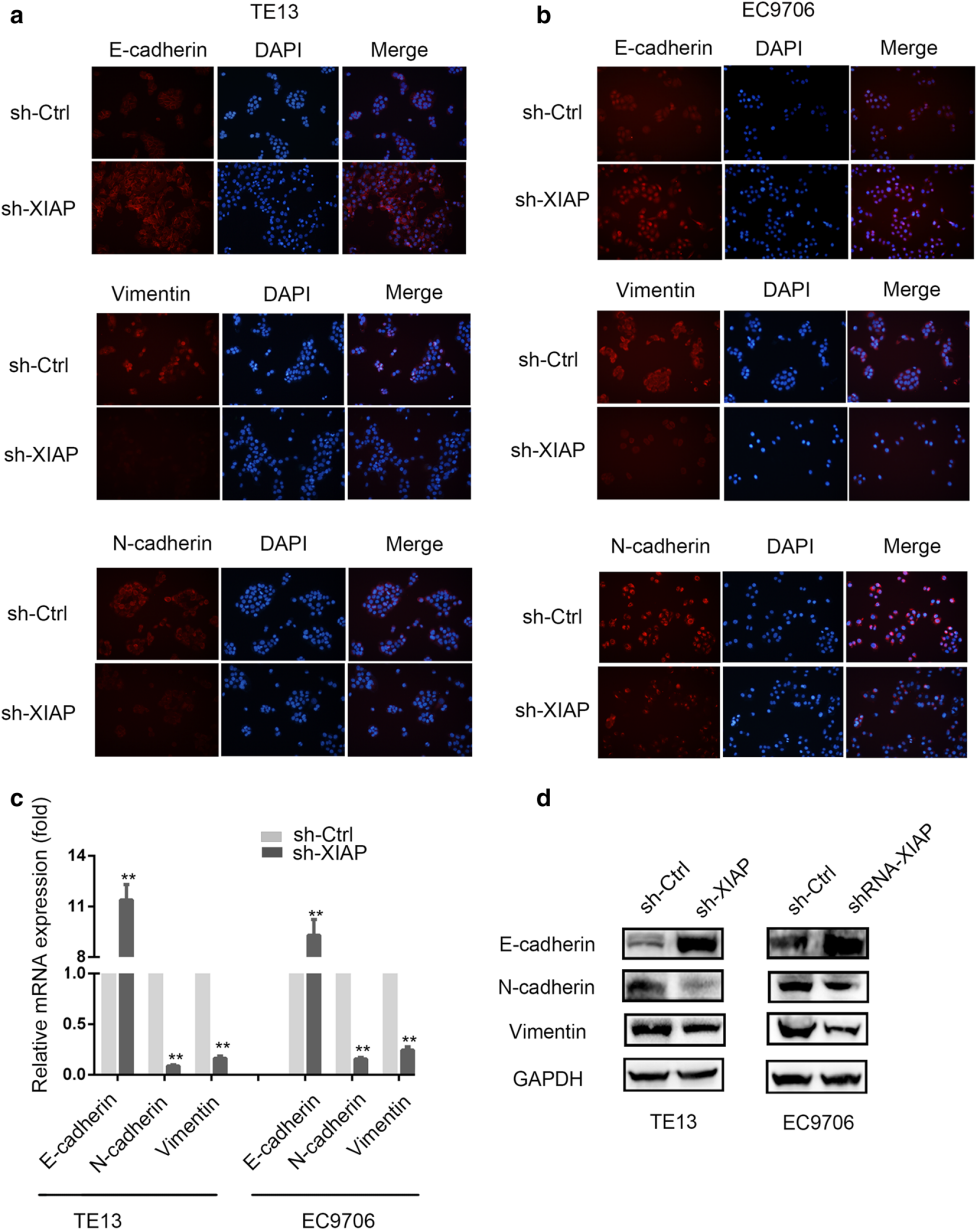
XIAP led EMT of ESCC cells. **a** E-cadherin, N-cadherin and Vimentin expression was performed by immunofluorescent staining, nuclei were counterstained with DAPI (×200) in TE13 cells. **b** E-cadherin, N-cadherin and Vimentin expression was performed by immunofluorescent staining, nuclei were counterstained with DAPI (×200) in EC9706 cells. **c** qRT-PCR was used to detect changes in expression of EMT genes in TE13 and EC9706 cells, results presented represent mean ± SD (n = 3) (*p < 0.05, **p < 0.01). **d** Western blotting was used to detect the expression of E-cadherin, N-cadherin and Vimentin in TE13 and EC9706 cells

We have demonstrated that XIAP knockdown inhibited the migration of ESCC cells. Many studies have showed that EMT plays a key role in the metastatic activity of cancers. In order to further investigate whether XIAP were exerting their migration effects through induction of EMT, We tested the expression levels of EMT markers including E-cadherin, N-cadherin and Vimentin in sh-XIAP and sh-Ctrl groups in TE13 and EC9706 cells respectively. As shown in Fig. 3a, b, epithelial marker (E-cadherin) was remarkably increased while the mesenchymal markers (N-cadherin and Vimentin) were decreased in sh-XIAP groups by immunofluorescence assay and also confirmed by qRT‑PCR and Western blotting. As shown in Fig. 3c, d, XIAP knockdown in ESCC cells led to up-regulate E-Cadherin and down-regulate N-Cadherin and Vimentin. These results validated the important role of XIAP on EMT induction.

### XIAP promoted lung metastasis and EMT of ESCC cells in vivo nude mice

**Fig. 4:**
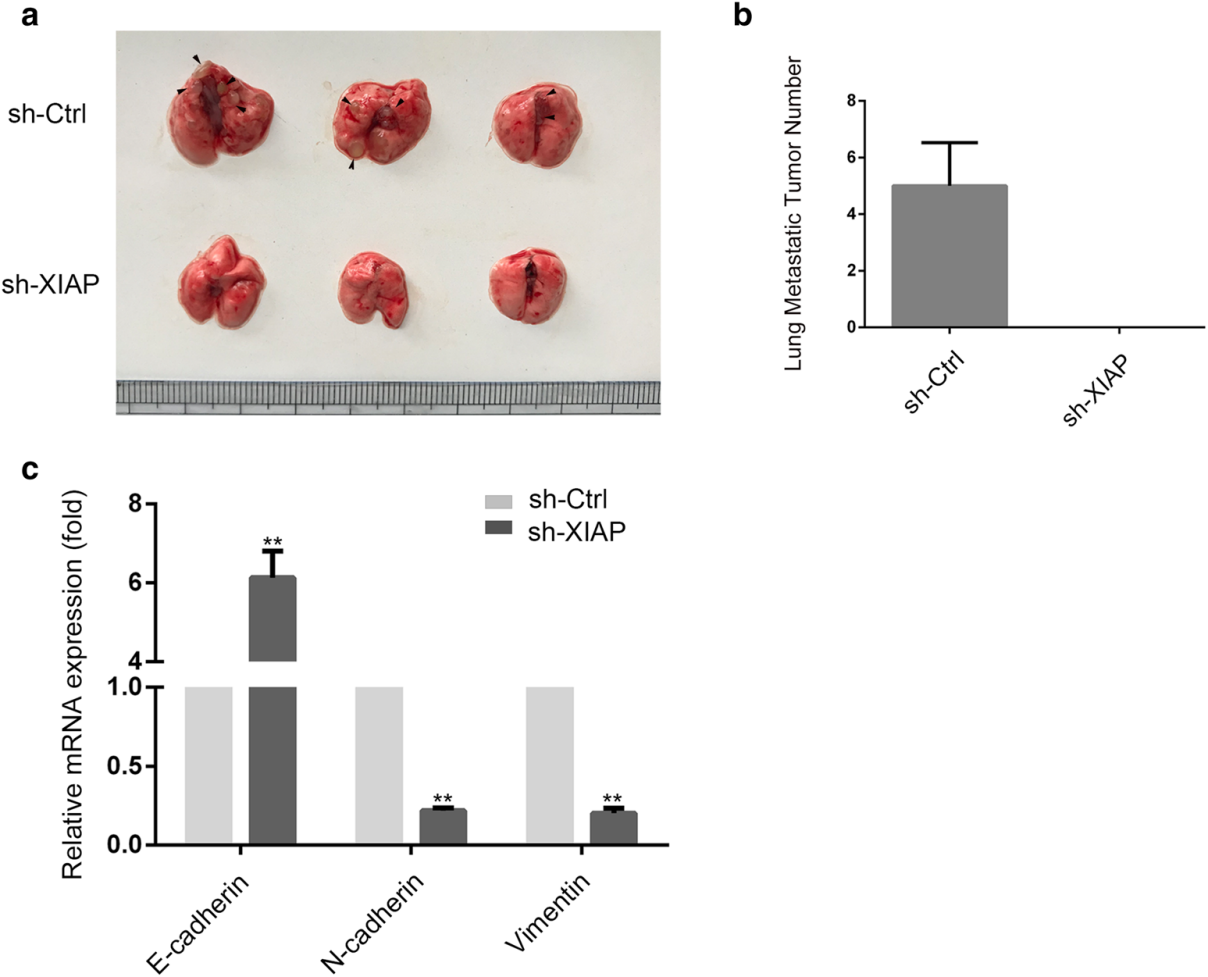
XIAP was essential for promoting EC9706 cell lung metastasis and EMT in vivo. **a** Representative images of the lungs and lung surface metastatic foci as indicated were shown in sh-Ctrl and sh-XIAP groups. **b** The lung metastatic tumor numbers of sh-Ctrl and sh-XIAP groups (*p < 0.05). **c** qRT-PCR was used to detect changes in expression of EMT genes in lung metastatic tumors, results presented represent mean ± SD (n = 3) (*p < 0.05, **p < 0.01)

The number of lung metastatic tumors in mice injected with EC9706 (sh-Ctrl) cells was remarkably increased than sh-XIAP group (Fig. 4a, b) (p < 0.01). In addition, as shown in Fig. 4c, epithelial marker (E-cadherin) was remarkably increased while the mesenchymal markers (N-cadherin and Vimentin) were decreased in sh-XIAP groups by qRT‑PCR. The results showed that XIAP is crucial for EC9706 cell lung metastasis and has an important role on EMT induction in vivo.

### XIAP knockdown decreased EMT through inhibiting TGF-β signaling pathway in ESCC cells

**Fig. 5:**
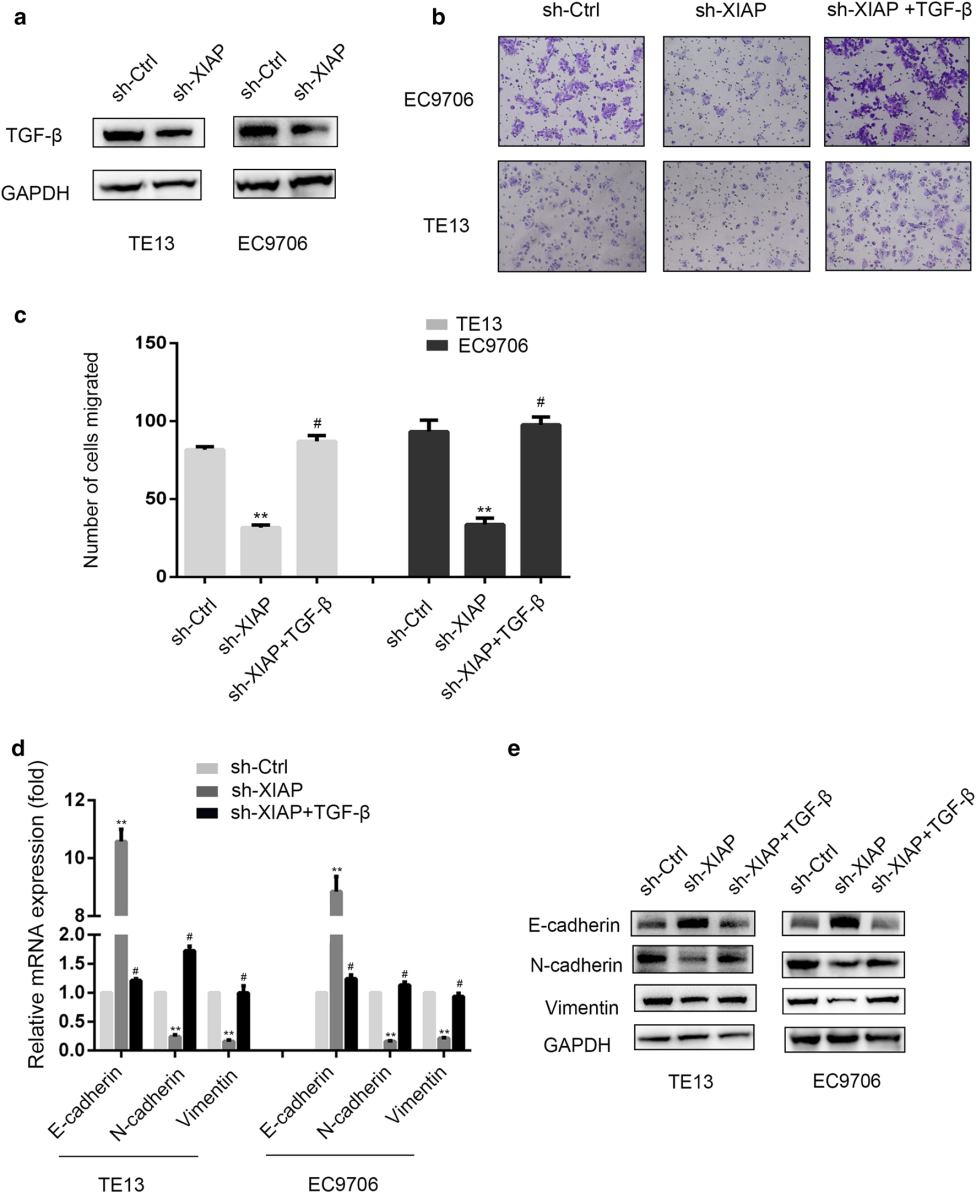
XIAP induced EMT by targeting TGF-β signal pathway in ESCC cells. **a** Western blotting was used to detect the expression of TGF-β in TE13 and EC9706 cells. **b** Microscopic pictures of Transwell assay for cell migration (×200). **c** Statistical chart of the number of migrated TE13 and EC9706 cells (*p < 0.05, **p < 0.01). **d** Expression of EMT genes was detected by qRT-PCR (**p < 0.01 versus sh-Ctrl group; ^#^p < 0.01 versus sh-XIAP group). **e** Western-blot was used to detect the expression of E-cadherin, N-cadherin and Vimentin with or without TGF-β

**Fig. 6:**
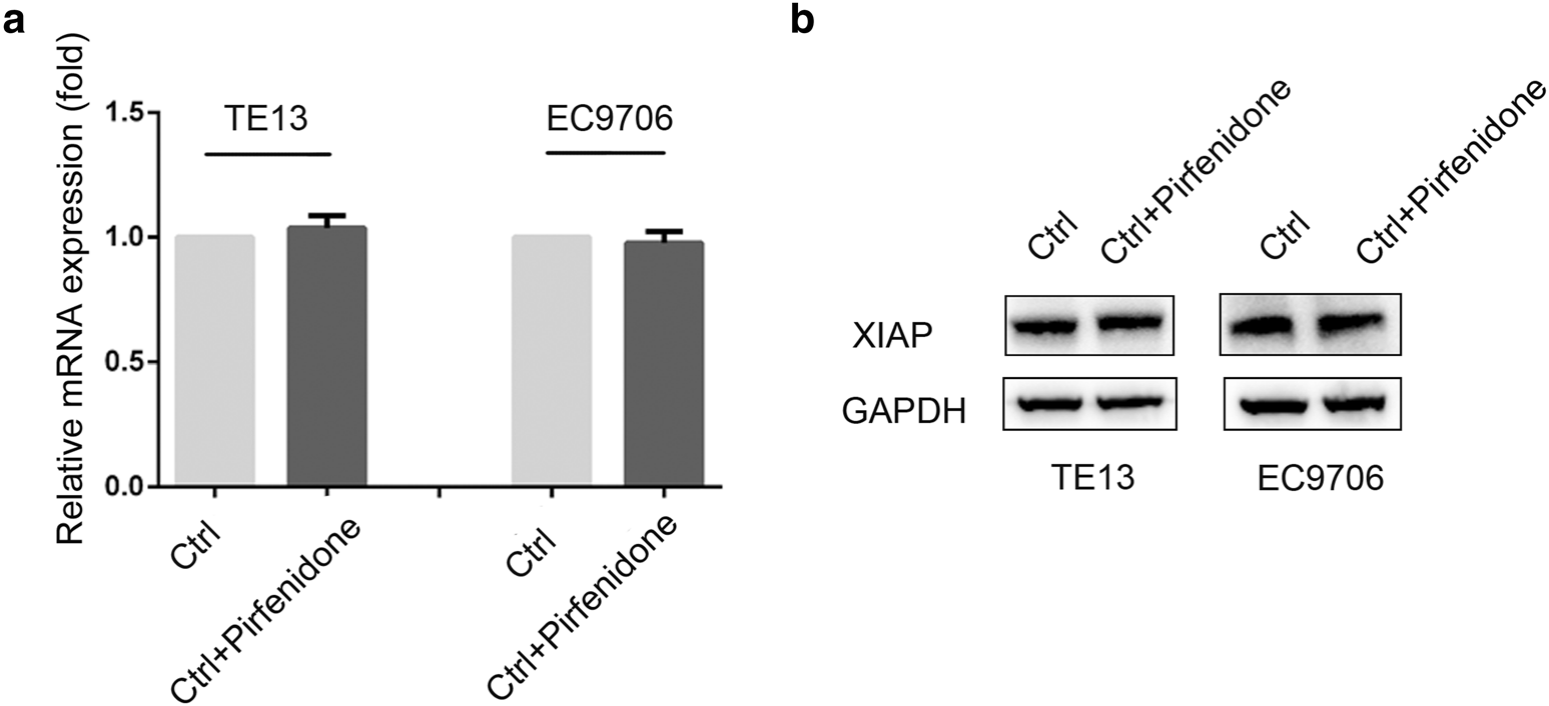
XIAP had no significant change in both ESCC cells when TGF-β signaling pathway was blocked. **a** qRT-PCR was used to detect the expression of XIAP in TE13 and EC9706 cells with TGF-β inhibitor (Pirfenidone) treatment. **b** Western blotting was used to examine the expression of XIAP with TGF-β inhibitor (Pirfenidone) treatment. Results are represented as mean ± SD

XIAP knockdown led to up-regulate E-Cadherin and down-regulate N-Cadherin and Vimentin. TGF-β is a key cytokine that mediates tumor metastasis through the activation of EMT. We questioned whether XIAP may regulate TGF-β and further enhance the EMT signaling pathway in ESCC cells. Firstly, we tested the expression levels of TGF-β by Western blotting and found that XIAP knockdown groups expressed low level of TGF-β (Fig. 5a). Then, XIAP knockdown groups were treated with exogenous TGF-β. Moreover, we used Transwell assay to evaluate the ability of migration in TE13 and EC9706 cells. The number of cells treated with exogenous TGF-β exhibited significantly increased compared with sh-XIAP groups (Fig. 5b, c). Furthermore, We examined effect of TGF-β in XIAP knockdown groups on EMT of ESCC cells by qRT‑PCR and Western blotting. As shown in Fig. 5d, e, TGF-β up-regulation led to down-regulate E-cadherin, and up-regulate Vimentin, N-cadherin compared with sh-XIAP groups. These results demonstrated that XIAP knockdown suppressed EMT of ESCC cells depending on TGF-β signaling. In order to further determine the potential role of XIAP in regulating TGF-β induced EMT, Pirfenidone, a TGF-β inhibitor was used to block TGF-β signaling. Then XIAP expression in TE13 and EC9706 cells was examined. The results indicated that XIAP had no significant change in both ESCC cell lines when TGF-β signaling pathway was blocked (Fig. 6a, b). Taken together, our results demonstrated that XIAP knockdown could suppress TGF-β induced EMT in ESCC cells.

### Correlation between XIAP expression and TGF-β expression in ESCC tissues

**Fig. 7:**
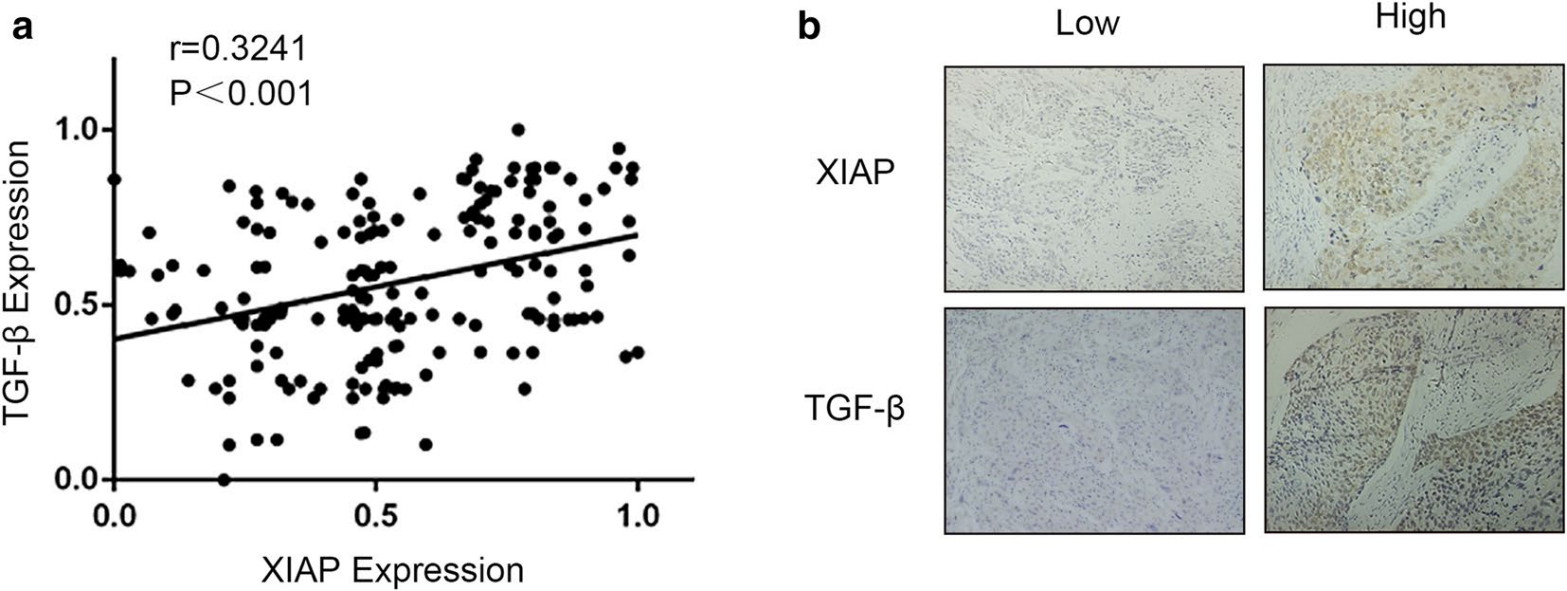
XIAP and TGF-β experssion in ESCC tissues. **a** Scatterplots with fitting line show positive correlation between XIAP and TGF-β expression (IOD) in ESCC tissues. Spearman correlation provides correlation coefficient (r) and p value. **b** Representative image of ESCC tissues in XIAP and TGF-β group by IHC staining (×200)

We used immunohistochemistry to detect the expression levels of XIAP and TGF-β in 185 ESCC tissues, and the two groups of continuous variables (IOD value) did not conform to the normal distribution, Spearman nonparametric correlation analysis was adopted. The results showed a positive correlation between XIAP and TGF-β expression in ESCC tissues (r = 0.3241, p < 0.001) (Fig. 7a, b). It was further verified that XIAP could promote invasion and metastasis of ESCC by upregulation of TGF-β expression.

## Discussion

ESCC is one of the most aggressive tumors with a high probability of metastasis, which leads to unfavorable prognosis after potentially curative treatment [19]. According to previous studies, XIAP plays a key role in many cancers and associated with poor prognosis [20, 21]. In this study, from 185 cases of human ESCC tissue samples, we investigated the correlation of XIAP with clinicopathologic characteristics of ESCC patients. Kaplan–Meier analysis indicated that ESCC patients with high XIAP expression in general had worse prognosis than those with low XIAP expression. Univariate and multivariate Cox regression analysis indicated that high XIAP expression was an independent risk factor for the prognosis of ESCC patients. The clinical data also showed that high level of XIAP was correlated with lymphatic metastasis.

We further questioned whether XIAP was related to migration of ESCC cells. XIAP was identified as a tumor activator because of its role on proliferation and apoptosis in cancer cells [22]. Furthermore, recent studies have shown a novel role of XIAP in regulating cancer cell motility varies upon cancer types [23]. Specifically, most of the discrepancy observed with respect to pro- and anti-migratory effects of XIAP, as many of the migration-regulating molecules controlled by XIAP could promote or inhibit migration in a context-dependent manner [24–26]. In cervical cancer, XIAP were demonstrated to directly bind to C-RAF which belongs to the Ras effector proteins (part of the MAPK cascade) and resulted in conformational change of C-RAF. In this way, XIAP decreased MAPK activity and cell migration [27, 28]. However, recent evidences suggested that XIAP could increase migration of cancer cells by directly interacting with the Rho GDP dissociation inhibitor (RhoGDI) via its RING domain [24, 29]. In response to a Wnt signal, XIAP was recruited to mono-ubiquitinates TLE and liberated TCF to form transcription complexes with β-catenin to active Wnt signal which was a key step in activate cell migration [30]. Here we further defined the effect of XIAP on ESCC migration ability. The results showed that XIAP knockdown could suppress the ESCC migration compared with control group. Previous study suggested that EMT has an important role in cancer cell migration and metastasis [15]. We demonstrated that XIAP is a crucial active factor in EMT. Knockdown of XIAP could change the expression of several typical EMT markers, including up-regulation epithelial marker (E-cadherin) and down-regulation of mesenchymal markers (N-cadherin and Vimentin). We also found that XIAP could promote lung metastasis and EMT of ESCC cells in vivo nude mice.

TGF-β has been shown to be an important regulator of cellular function, such as increased tumorigenesis, invasiveness, and cell migration [31]. Furthermore, TGFβ is also a critical cytokine that mediates tumor metastasis through the activation of EMT [32–34]. To explore the potential mechanisms involved in the function of XIAP, we investigated whether XIAP regulates TGFβ-induced EMT. As expected, our results suggested that TGFβ-induced EMT could be depressed by inhibit XIAP in ESCC. Furthermore, we questioned whether TGFβ might regulate the expression of XIAP. Our results showed that XIAP had no significant change in both ESCC cell lines when TGF-β signaling pathway was blocked.

## Conclusion

In conclusion, the present study revealed that XIAP significantly correlated with a poor prognosis of ESCC. We also demonstrated that the critical role of XIAP in EMT through regulation of TGF-β signaling pathways. Therefore, our results may provide a new potential target for the treatment of ESCC.

## Supplementary information

**Additional file 1: Figure S1.** Generation of stable XIAP knockdown cell lines. (A) The level of mRNA expression in sh-Ctrl and sh-XIAP constructs was analyzed by qRT-PCR. (B) Western-blot was used to examine the expression of XIAP. (**p < 0.01 versus sh-Ctrl group).

## Data Availability

All relevant data are within this published paper.

## Abbreviations

ESCC: esophageal squamous cell cancer
XIAP: X-linked apoptotic inhibitory protein
EMT: epithelial-to-mesenchymal transition
qRT-PCR: quantitative real-time polymerase chain reaction
TGFβ: transforming growth factor-β
