# Supplementary figures and images for: X-linked inhibitor of apoptosis protein accelerates migration by inducing epithelial–mesenchymal transition through TGF-β signaling pathway in esophageal cancer cells
Source: Cell Biosci. 2019 Sep 18;9:76. doi: 10.1186/s13578-019-0338-3 (PMC6749643; doi:10.1186/s13578-019-0338-3)

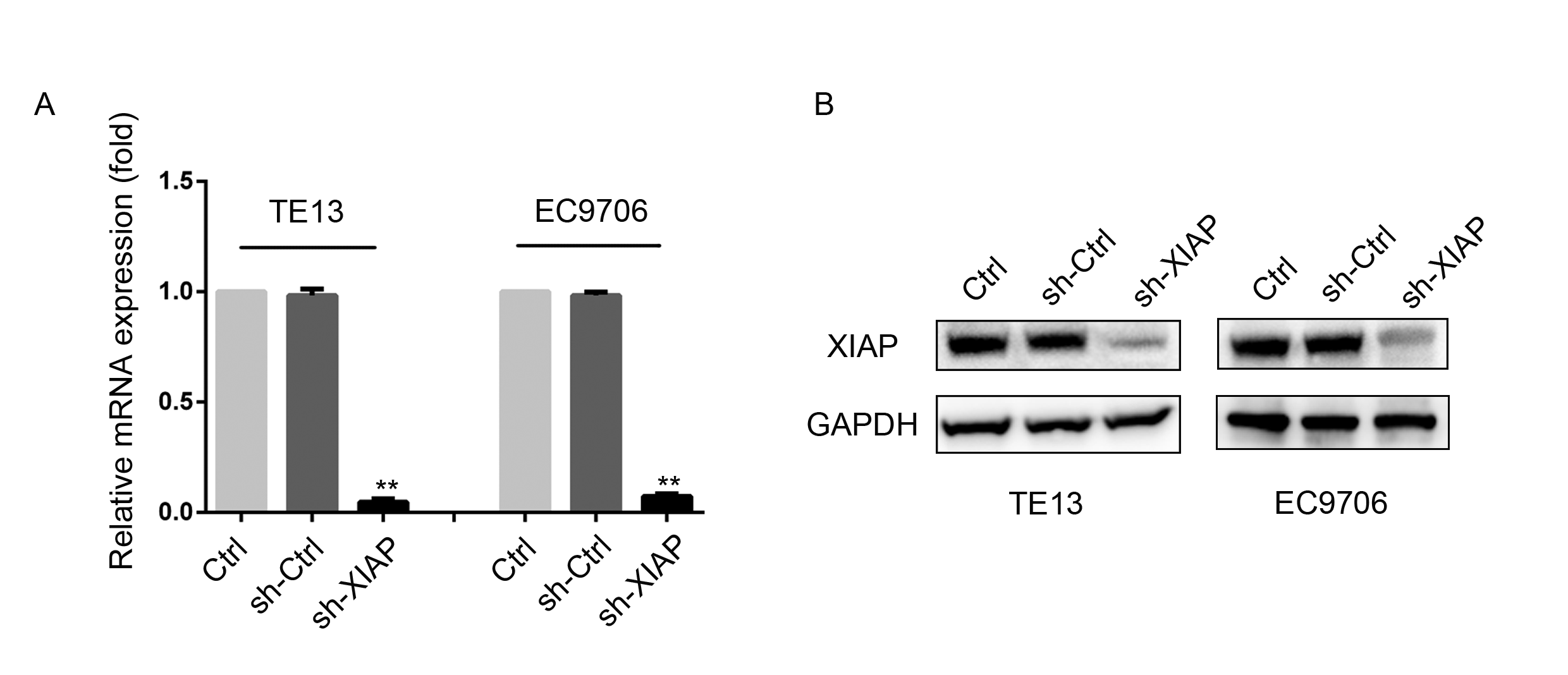

Supplement: Supplementary file 1 — Additional file 1: Figure S1. Generation of stable XIAP knockdown cell lines. (A) The level of mRNA expression in sh-Ctrl and sh-XIAP constructs was analyzed by qRT-PCR. (B) Western-blot was used to examine the expression of XIAP. (**p < 0.01 versus sh-Ctrl group). [file 13578_2019_338_MOESM1_ESM.tif]
